# Effects of Preoperative Intraocular Pressure Level on Surgical Results of Microhook *Ab Interno* Trabeculotomy

**DOI:** 10.3390/jcm10153327

**Published:** 2021-07-28

**Authors:** Masaki Tanito, Kazunobu Sugihara, Aika Tsutsui, Katsunori Hara, Kaoru Manabe, Yotaro Matsuoka

**Affiliations:** 1 Department of Ophthalmology, Faculty of Medicine, Shimane University, Izumo 693-8501, Japan; ksugi@med.shimane-u.ac.jp (K.S.); aika0408@med.shimane-u.ac.jp (A.T.); hakari55@med.shimane-u.ac.jp (K.H.); 2 Division of Ophthalmology, Matsue Red Cross Hospital, Matsue 690-8506, Japan; manabe42@med.shimane-u.ac.jp (K.M.); ymatsu@med.shimane-u.ac.jp (Y.M.)

**Keywords:** minimally invasive glaucoma surgery (MIGS), Tanito microhook (TMH), surgical efficacy, surgical complication, review

## Abstract

To assess the roles of preoperative intraocular pressure (IOP) level on the IOP reducing efficacy of microhook *ab interno* trabeculotomy (µLOT), 126 consecutive open-angle glaucomatous eyes (90 primary open angle glaucoma, 36 exfoliation glaucoma) of 126 Japanese subjects (60 men, 66 women; mean age ± standard deviation, 70.5 ± 11.4 years) who underwent µLOT alone (25 eyes, 20%) or combined µLOT and cataract surgery (101 eyes, 80%) were retrospectively included, and subdivided into four groups based on the quartile of preoperative IOP: Q1, ≤15 mmHg; Q2, 15–18 mmHg, Q3, 18–21 mmHg, and Q4, >21 mmHg. Preoperative and 12 months postoperative IOPs and numbers of antiglaucoma medications were compared among IOP groups. Factors associated with postoperative IOP were assessed using multivariate analysis, and the success of IOP control was assessed with various definitions. Postoperatively, IOP was significantly higher in Q3 (*p* < 0.0146) and Q4 (*p* = 0.0320) groups than Q1 group, while the number of medications was not significantly different among four IOP groups (*p* = 0.1966). Older age was associated with lower postoperative IOP, and higher preoperative IOP was associated with higher postoperative IOP, while sex, glaucoma type, surgical procedure, lens status, extent of trabeculotomy incision, and preoperative number of medications were not associated with postoperative IOP. The success rates for IOP ≤ 18 and ≤15 mmHg were higher in lower preoperative IOP groups (i.e., Q1 and Q2) than higher preoperative IOP groups (Q3 and Q4), while the success rate for ≥20% IOP reduction was higher in higher preoperative IOP groups than in lower preoperative IOP groups; the success rate for ≥0% IOP reduction was equivalent among groups. By reviewing the previous studies in *ab interno* trabeculotomy/goniotomy procedures, positive correlation between preoperative and postoperative IOPs was preserved throughout the studies and surgical procedures. After the µLOT, larger %IOP reduction was achieved in higher preoperative IOP groups than in lower preoperative IOP groups, while postoperative IOP was still lower than in lower preoperative IOP groups.

## 1. Introduction

Trabeculotomy (LOT) lowers intraocular pressure (IOP) by reducing aqueous flow resistance by cleaving the trabecular meshwork (TM) and inner walls of Schlemm’s canal [1]. The blebless mechanism of IOP reducing action in LOT is associated with less chance of devastating visual complications including shallow anterior chamber (AC), bleb leakage, blebitis, choroidal detachment, and hypotony maculopathy than those in mitomycin C-adjuvanted trabeculectomy [2,3]. In recent years, *ab interno* approaches have been adopted for performing LOT procedures [4,5]. In 2015, as an initial case, we performed microhook trabeculotomy (µLOT), a novel *ab interno* LOT procedure, in a case with bilateral steroid-induced glaucoma [6]. Since a favorable result was obtained in that case, we treated the other cases with µLOT; we have reported surgical results and safety profiles in these cases [7,8,9]. In an initial 560 eyes, the preoperative IOP of 20.2 mmHg and number of antiglaucoma medications of 2.8 decreased to 13.9 mmHg (31% reduction) and 2.5 (11% reduction), respectively, at the mean final evaluation of 13.5 months after µLOT [9].

Previously, various factors that possibly associate with the surgical efficacy of LOT/goniotomy surgeries were reported; the factors assessed included age [10], preoperative IOP level [11,12,13], degree of angle opening [14], glaucoma severity [15,16,17,18], and simultaneous cataract surgery [10,19,20,21]. In case series of µLOT, by using multiple regression analyses, we have previously reported that older age, steroid-induced glaucoma, developmental glaucoma, and the absence of postoperative complications were associated with lower final IOP; exfoliation glaucoma, other types of glaucoma, and higher preoperative IOP were associated with higher final IOP [9]. Given the safety profiles of recent minimally invasive glaucoma surgeries (MIGS) [22], a growing number of *ab interno* LOT/goniotomy procedures are considered as treatment options in eyes with relatively low preoperative IOP, but the surgical efficacy by µLOT in lower IOP eyes has not been fully assessed.

In the current study, to test the effects of preoperative IOP levels on the IOP reducing efficacy of µLOT more precisely, the surgical results were compared between groups stratified by preoperative IOP levels.

## 2. Materials and Methods

### 2.1. Methods

This retrospective study included 126 consecutive glaucomatous eyes of 126 Japanese subjects (60 men, 66 women; mean age ± standard deviation [SD], 70.5 ± 11.4 years) who underwent µLOT performed by one surgeon (M.T.) at Matsue Red Cross Hospital between May 2015 and March 2018 to control the IOP. Among the 560 eyes of 375 patients who were filed in the department’s database [9], the subjects with open-angle glaucoma (primary open-angle glaucoma (POAG) or exfoliation glaucoma (EXG)), without previous ocular surgical history, other than small incisional cataract surgery, and who followed up for longer than 12 months were chosen for this study. If both eyes of a subject were eligible, the eye upon which µLOT was performed earlier was included. The study adhered to the tenets of the Declaration of Helsinki; the institutional review board (IRB) of Matsue Red Cross Hospital reviewed and approved the research (IRB No. 261). Preoperatively, all subjects provided written informed consent for surgery and use of the clinical data regarding the glaucoma treatment obtained during the follow-up periods. Based on the quartile levels of preoperative IOP, the eyes were subdivided into four groups: Q1, ≤15 mmHg; Q2, 15–18 mmHg, Q3, 18–21 mmHg, and Q4, >21 mmHg. The patients’ demographic data and surgical procedures are summarized in Table 1.

### 2.2. Surgical Procedure

µLOT was performed as described previously [7,8]. Three specifically designed microhooks for µLOT, i.e., straight (M-2215S), right-angled (M-2215R), and left-angled (M-2215L) (all from Inami & Co., Ltd., Tokyo, Japan), were used [23]. When the combined procedure was performed, phacoemulsification cataract surgery was performed before µLOT; the cataract surgery was performed through a 2.2-mm-wide clear corneal incision created at the 9 to 10 o’clock position (i.e., temporal incision for the right eye and nasal incision for the left eye) and a corneal port created at the 2 to 3 o’clock position. A one-piece soft-acrylic intraocular lens (IOL) was inserted through the same clear corneal incision; the Vivinex iSert XY1 IOL (Hoya, Tokyo, Japan) was used in most cases, and the AcrySof IQ IOL (Alcon Japan, Tokyo, Japan) and Tecnis OptiBlue IOL (AMO Japan, Tokyo, Japan) in others. After IOL implantation, standard sub-Tenon anesthesia was induced using 2% lidocaine (in most earlier cases) or intracameral anesthesia using 1% lidocaine (in most later cases). A viscoelastic material (1% sodium hyaluronate, Opegan Hi, Santen Pharmaceutical, Osaka, Japan) was injected into the AC to widen the angle. Using a Swan-Jacob gonioprism lens (Ocular Instruments, Bellevue, WA) to observe the angle, a microhook was inserted into the AC through the corneal incision. The tip of the microhook then was inserted into Schlemm’s canal and moved circumferentially to incise the inner wall of Schlemm’s canal and TM beyond the 3 o’clock position. Using the same procedure, LOT was performed in the opposite angle using a microhook that was inserted through the corneal port. Accordingly, beyond the 6 o’clock position, the TM was incised when both nasal and temporal angles were operated on. To improve the operability in most cases, a straight hook was used to incise the nasal angle, and the right-angled and left-angled hooks were used to incise the temporal angle. After the viscoelastic material was aspirated, the corneal incision and port were closed by corneal stromal hydration. At the end of surgery, 1.65 mg of dexamethasone sodium phosphate (Decadron, Aspen Japan, Tokyo, Japan) was injected subconjunctivally and 0.3% ofloxacin ointment (Tarivid, Santen Pharmaceutical) was applied. Finally, 1.5% levofloxacin (Nipro, Osaka Japan) and 0.1% betamethasone (Sanbetason, Santen Pharmaceutical) were applied topically four times daily for 3 to 4 weeks (i.e., 1 bottle/eye) postoperatively in all cases. Topical non-steroidal anti-inflammatory drugs were not used routinely.

### 2.3. Measurements

The clinical parameters, including age, sex, glaucoma type, lens status, ocular surgical history, and surgical procedure (i.e., µLOT alone or combined µLOT and cataract surgery), extent of trabeculotomy, surgical complications and interventions were collected from the medical charts. Preoperative and 12 months (range, 11–14 months) postoperative IOP and number of antiglaucoma medications also were collected, and %IOP reduction (preoperative IOP minus postoperative IOP) and %medication reduction (preoperative medication number minus postoperative medication number) were calculated. The IOP was measured using Goldmann applanation tonometry.

### 2.4. Statistical Analysis

For continuous variables, one-way analysis of variance (ANOVA) was used for the comparison of 4 IOP groups; when ANOVA was significant, the Tukey–Kramer honesty significant difference test was used for each pair comparison. For categorical variables, the exact Cochrane–Armitage trend test was used for the comparison of the 4 groups. In each group, pre- and post-operative values were compared by using the paired t-test. Possible factors that associate with 12 months postoperative IOP were assessed by multiple regression analysis. To assess the effect of preoperative IOP on 12 months postoperative IOP level, IOP control was calculated by postoperative IOP ≤18, ≤15, and ≤12 mmHg, or IOP reduction ≥20% and ≥0%, and a combination of these definitions, separately. All continuous data were expressed as the mean ± SD. All statistical analyses were performed using the JMP version 11.0 statistical software (SAS Institute, Inc., Cary, NC, USA). *p* < 0.05 was considered significant. For 4-group comparisons of %IOP reduction, when the alpha error = 0.05, standard deviation = 17%, and the mean %IOP reduction in each group = 17.7%, 28.8%, 28.2%, and 46.7%, the statistical power was calculated to be 0.78 in this dataset.

## 3. Results

Table 1 summarizes the patient data. EXG was more frequent in higher preoperative IOP groups than lower preoperative IOP groups (*p* < 0.0001), while other parameters, including age, sex, lens status, surgical procedure (i.e., µLOT alone or combined µLOT), trabeculotomy sites, and extent of trabeculotomies, were equivalent among IOP groups.

In all IOP groups, compared with preoperative IOP, postoperative IOP was significantly lower at 12 months (*p* < 0.0001 in all comparisons) (Table 2). Preoperatively, IOP was different between every comparison pair among Q1-Q4 IOP groups (*p* < 0.0001–0.0366). At 12 months postoperatively, IOP was significantly higher in Q3 (*p* < 0.0146) and Q4 (*p* = 0.0320) groups than Q1 group. The %IOP reduction was significantly different among all comparison pairs of Q1-Q4 groups (*p* < 0.0001–0.0493), except for the comparison between Q2 and Q3 groups (*p* = 0.9990). In Q1, Q2, and Q3 groups, the number of glaucoma medications was significantly lower postoperatively than preoperatively (*p* = 0.0180–0.0392). Preoperative (*p* = 0.2499) and postoperative (*p* = 0.1966) numbers of medication, and %medication reduction (*p* = 0.9063) were not significantly different among the four IOP groups.

Intraoperative complications and additional procedures were recorded in five (4%) eyes and three (2%) eyes, respectively (Table 3). Postoperative complications developed and interventions were required in 57 (45%) eyes and 16 (13%) eyes, respectively (Table 3). The most common postoperative complications and interventions were layered hyphema in 42 (33%) eyes and hyphema washout in 9 (7%) eyes, respectively.

**Figure 1:**
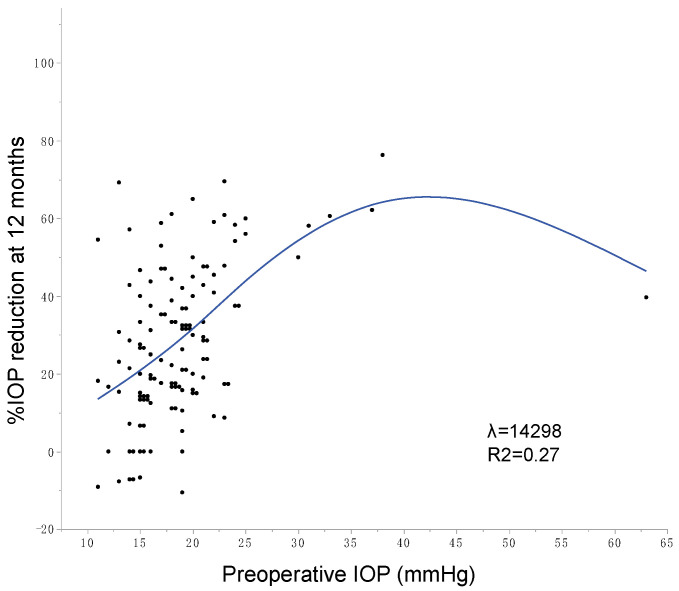
Correlation between preoperative and postoperative 12 months IOP. Blue line and area indicate spline fit (λ = 14928, R^2^ = 0.27, sum of squared errors of prediction = 34915).

The possible factors associated with the 12 months postoperative IOP were assessed by multiple regression analyses (Table 4). Among the factors included in the model, older age was associated with lower postoperative IOP, and higher preoperative IOP was associated with higher postoperative IOP, while sex, glaucoma type (POAG or EXG), surgical procedure (µLOT alone or combined µLOT), lens status (phakic or pseudophakic), extent of incision, and preoperative number of medications were not associated with postoperative IOP. In the scatter plots, except for the extreme cases (i.e., preoperative IOP <12 mmHg or >30 mmHg), virtually linear association between preoperative IOP and %IOP reduction is observed in our cases (Figure 1).

The success rates of IOP control defined by absolute IOP levels and %IOP reduction in each preoperative IOP group are summarized in Table 5. The success rates for IOP ≤ 18 and ≤15 mmHg were higher in lower preoperative IOP groups (i.e., Q1 and Q2) than higher preoperative IOP groups (Q3 and Q4), while the success rate for ≥20% IOP reduction was higher in higher preoperative IOP groups than lower preoperative IOP groups. As a result, the success rate of IOP control was significantly higher in higher preoperative IOP groups than lower preoperative IOP groups when the success was defined by combination of absolute IOP (i.e., ≤18 or ≤15 mmHg) and ≥20% IOP reduction. The success rates were not statistically different among groups when the success was determined by the definitions including ≥0% IOP reduction.

## 4. Discussion

**Figure 2:**
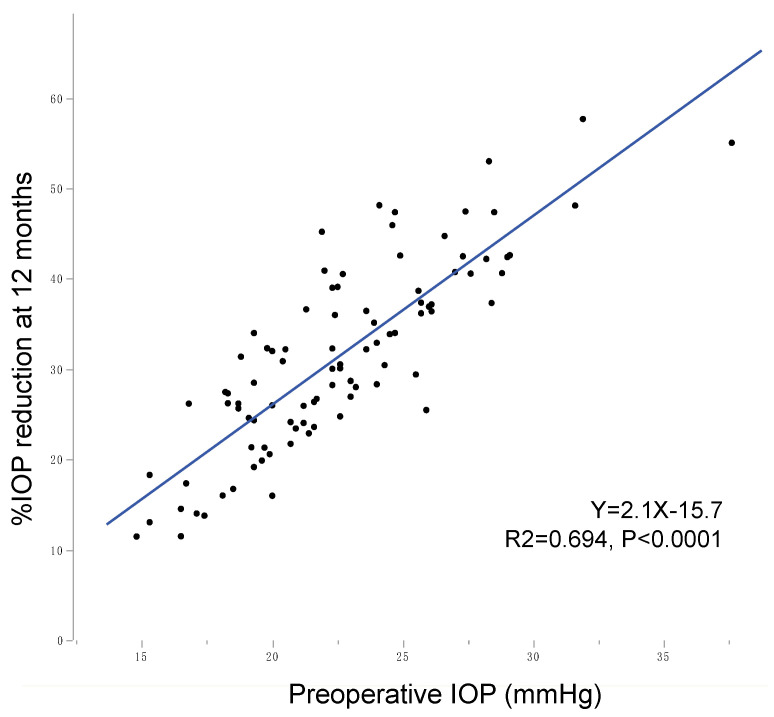
Correlation between preoperative and postoperative 12 months IOP after various *ab interno* goniotomy procedures in the published literature. A full list of studies is found in Supplementary Table S1. Blue line and area indicate linear regression and 95% confidence intervals, respectively.

**Figure 3:**
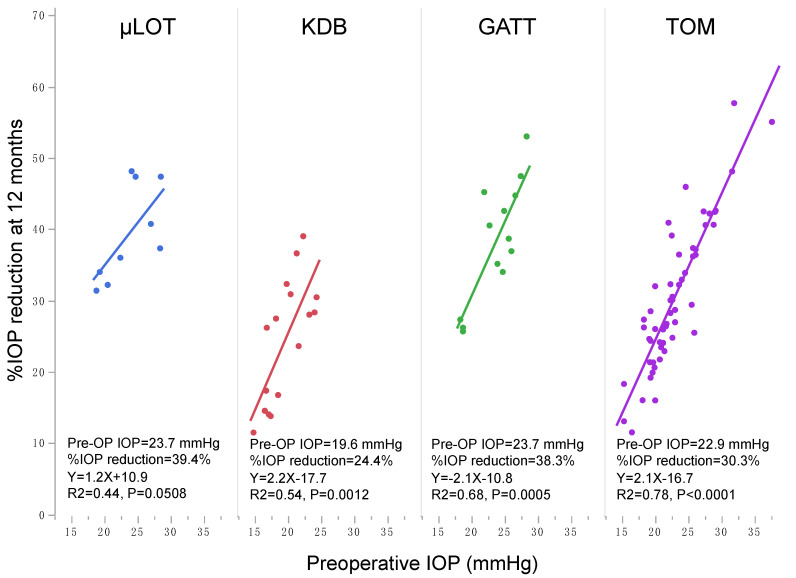
Correlation between preoperative and postoperative 12 months IOP after each *ab interno* goniotomy procedure in the published literature. Each line and area indicate linear regression and 95% confidence intervals, respectively. µLOT, microhook trabeculotomy; KDB, Kahook dual blade; GATT, gonioscopy-assisted transluminal trabeculotomy; TOM, Trabectome.

**Figure 4:**
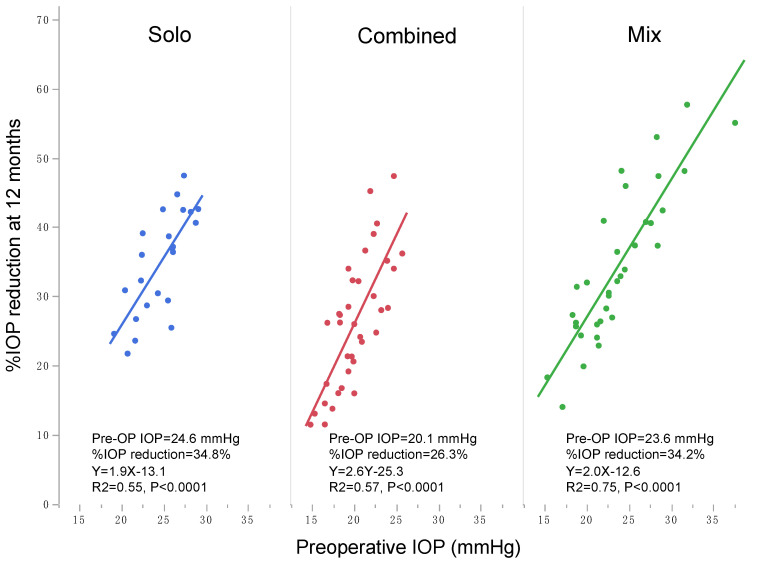
Correlation between preoperative and postoperative 12 months IOP after solo, combined, or both solo and combined *ab interno* goniotomy procedures in the published literature. Each line and area indicate linear regression and 95% confidence intervals, respectively.

In this study, although the statistically significant reduction in IOP was observed in each IOP group, their magnitudes were remarkably different, i.e., larger %IOP reduction was achieved in higher preoperative IOP groups than lower preoperative IOP groups (Table 2). Using multiple regression analysis, preoperative IOP was the significant indicator with the highest standard β value for predicting postoperative IOP (Table 4). A lower IOP-reducing magnitude in the lower preoperative IOP group than in the higher IOP group was reported after other goniotomy procedures with Kahook dual blade (KDB) [11,12] and trabectome [13]. Figure 2 shows the correlation between preoperative IOP and 12 months %IOP reduction after various goniotomy/LOT surgeries including µLOT, KDB, gonioscopy-assisted transluminal trabeculotomy (GATT), and trabectome in subject groups including POAG (data and references used for generation of this figure are shown in Supplementary Table S1). The figure clearly depicts the linear correlation between preoperative IOP level and postoperative %IOP reduction. This is true when the correlation was tested in each surgical procedure separately (Figure 3) or in each solo, combined, or mixture of solo and combined procedure separately (Figure 4). Correctively to the previous studies, our results further confirm the roles of preoperative IOP on postoperative IOP achieved by *ab interno* goniotomy procedures. The reduction mechanism of goniotomy procedures—that is, the re-establishment of Schlemm’s canal outflow pathway by the elimination of TM resistance—should be limited by the remaining resistance existing distal to collector channels [24], and thus the floor effects might explain the reduced IOP reduction in eyes with lower preoperative IOP observed in this and previous studies.

In this study, older age was associated with a lower postoperative IOP level. Previously, older age was associated with higher success rates of IOP control at less than 17 and 15 mmHg after *ab externo* LOT [10]. With aging, TM resistance increases [25], while aqueous humor production decreases [26]; when the preoperative IOP levels were equal between young and old age groups, the effects of elimination of TM resistance on IOP reduction should be greater in older subjects than in younger subjects. Accordingly, this can be an explanation of the negative correlation between age and postoperative IOP in this study. In experimental studies [27,28,29], the decreased outflow resistance after LOT might be caused by direct communication between Schlemm’s canal and the anterior chamber at an early postoperative stage. Subsequently, the repair process of trabecular tissue, occurring initially in the corneoscleral and endothelial meshwork and finally in the uveal meshwork, causes increments in resistance to aqueous outflow [27,28,29]. In general, inflammatory reactions become mild with aging, and so wound healing may be retarded in older subjects. Accordingly, a weak repair process at the trabeculotomy site due to an impaired healing reaction is another possibility for lower postoperative IOP in older patients.

Although the % reduction was smaller in lower preoperative IOP groups, postoperative IOP was still lower in lower IOP groups than in higher IOP groups. This explains the reversal of surgical success rates among IOP groups, i.e., higher probability of success in lower IOP groups when the success was defined by absolute postoperative IOP values, while there was a higher probability of success in higher IOP groups when the success was defined by %IOP reductions (Table 5). Currently, in eyes with early to moderate glaucoma with a visually significant cataract, combined cataract and MIGS including µLOT can be a candidate surgical procedure; the purpose of surgery can sometimes be a reduction in medication number or a modest reduction in IOP. In this scenario, not achieving ≥20% IOP reduction may not be unsuccessful for both patients and surgeons. Although the combined use of absolute IOP levels (i.e., 12, 15, 18, or 21 mmHg) and %IOP reduction (i.e., 20% or 30%) has been recommended to report the efficacy of glaucoma surgery [30], this type of definition might underestimate the merit of MIGS, especially when the procedure is performed in eyes with low preoperative IOP. Our results show that the trend of lower success rates in lower preoperative IOP groups disappeared when ≥0%IOP reduction was included in the definition of success, thus the combined use of each absolute IOP level with the IOP not exceeding preoperative IOP level might be suitable to avoid such underestimation, but this requires further study to build consensus.

Various complications developed perioperatively (Table 3), although most resolved spontaneously or were treated with relatively minor interventions such as washout of the hyphema. In our dataset, the rates of postoperative complications such as layered hyphema formation were not remarkably different among IOP groups (data not shown, *p* = 0.07844 by G-test). The limitations of the current study included the retrospective design and relatively short follow-up. The inclusion of both eyes with combined and solo procedures can be a selection bias, although the rates of solo/combined procedures were equivalent among IOP groups, and different procedures were adjusted by the multivariate analyses. In this study, no additional IOP reduction by combined cataract surgery was detected (Table 4). This is in line with the previous reports in trabectome [20,21]; however, it disagrees with our previous study in *ab externo* trabeculotomy [31]. Since the current study is not specifically designed to test the efficacy of cataract surgery on IOP reduction, further study is required to conclude on the additive effect of cataract surgery on IOP in µLOT. Despite the several weak points, we believe that our study design is reasonable to the evaluate the effects of preoperative IOP levels on surgical efficacy of µLOT, and the review results of previous evidence seem to allow us to generalize our observations to other goniotomy procedures.

## 5. Conclusions

In summary, after the µLOT, larger %IOP reduction was achieved in higher preoperative IOP groups than in lower preoperative IOP groups, while postoperative IOP was still lower than in lower preoperative IOP groups.

## Figures and Tables

**Table 1:** Demographic patient data.

Parameters	Total	Q1	Q2	Q3	Q4	*p* Value ^†^
Preoperative IOP		≤15 mmHg	>15 mmHg and ≤18 mmHg	>18 mmHg and ≤21 mmHg	>21 mmHg	
Eyes/Subjects	126/126	38/38	30/30	36/36	22/22	
Age, years	70.5 ± 11.4 (33, 88)	71.2 ± 12.0 (33, 88)	70.7 ± 12.3 (34, 87)	67.6 ± 11.6 (38, 86)	74.0 ± 8.0 (56, 85)	0.2046
Sex, subjects (%)						
Male	60 (48)	14 (37)	17 (57)	14 (39)	15 (68)	0.0998
Female	66 (52)	24 (63)	13 (43)	22 (61)	7 (32)	
Glaucoma type, eyes						
POAG	90 (71)	34 (89)	26 (87)	22 (61)	8 (36)	<0.0001 **
EXG	36 (29)	4 (11)	4 (13)	14 (39)	14 (64)	
Lens status, eyes						
Phakia	118 (94)	36 (95)	29 (97)	33 (92)	20 (91)	0.7312
Pseudophakia	8 (6)	2 (5)	1 (3)	3 (8)	2 (9)	
Surgical procedure, eyes						
µLOT alone	25 (20)	6 (16)	7 (23)	8 (22)	4 (18)	0.4315
µLOT + cataract surgery	101 (80)	32 (84)	23 (77)	28 (78)	18 (22)	
Trabeculotomy site, eyes						
Nasal and temporal	111 (88)	32 (84)	25 (83)	33 (92)	21 (95)	
Nasal only	4 (3)	2 (5)	0 (0)	1 (3)	1 (5)	
Temporal only	11 (9)	4 (11)	5 (17)	2 (6)	0 (0)	
Extent of trabeculotomies, clock hours						
Nasal and temporal	6.9 ± 0.9 (5, 9)	6.8 ± 0.9 (5, 8)	7.0 ± 1.0 (5, 9)	6.8 ± 0.8 (5, 8)	6.9 ± 0.9 (5, 9)	
Nasal only	3.8 ± 0.5 (3, 4)	3.5 ± 0.7 (3, 4)		4	4	
Temporal only	3.6 ± 0.7 (3, 5)	3.8 ± 0.5 (3, 4)	3.2 ± 0.4 (3, 4)	4.5 ± 0.7 (4, 5)		

Data are expressed in mean ± SD (range) for continuous data and no. (%) for categorical data. ^†^ *p* values are calculated using one−way analysis of variance (ANOVA) for continuous data and the exact Cochrane−Armitage trend test for categorical data among 4 groups stratified by preoperative intraocular pressure level. ** indicate significance level of 1%, respectively. Abbreviations: IOP, intraocular pressure; POAG, primary open-angle glaucoma; EXG, exfoliation glaucoma; µLOT, microhook *ab interno* trabeculotomy.

**Table 2:** Preoperative and 12 months postoperative IOP and medications.

Parameters	Total	Q1	Q2	Q3	Q4	*p* Value ^†^
IOP						
Pre-op, mmHg	18.8 ± 6.0 (11, 63)	14.0 ± 1.3 (11, 15)	17.2 ± 0.9 (16, 18)	19.8 ± 0.9 (19, 21)	27.5 ± 9.3 (22, 65)	<0.0001 **
*p*-value ^‡^, vs. ≤15 mmHg group			0.0092 **	<0.0001 **	<0.0001 **	
*p*-value ^‡^, vs. >15 and ≤18 mmHg group				0.0366 *	<0.0001 **	
*p*-value ^‡^, vs. >18 and ≤21 mmHg group					<0.0001 **	
12 M post-op, mmHg	12.9 ± 3.9 (4, 38)	11.6 ± 2.9 (4, 16)	12.2 ± 2.7 (7, 16)	14.2 ± 2.8 (7, 21)	14.3 ± 6.5 (7, 38)	0.0048 **
*p*-value ^‡^, vs. ≤15 mmHg group			0.8920	0.0146 *	0.0320 *	
*p*-value ^‡^, vs. >15 and ≤18 mmHg group				0.1379	0.1833	
*p*-value ^‡^, vs. >18 and ≤21 mmHg group					0.9993	
Difference, mmHg	5.9	2.4	5.0	5.6	13.2	
*p* value ^#^, pre- vs. post-op	<0.0001 **	<0.0001 **	<0.0001 **	<0.0001 **	<0.0001 **	
%IOP reduction, %	28.4 ± 19.5 (−10.5, 76.3)	17.7 ± 19.2 (−9.1, +69.2)	28.8 ± 15.5 (0, 61.1)	28.2 ± 14.9 (−10.5, 65)	46.7 ± 19.0 (8.7, 76.3)	<0.0001 **
*p*-value ^‡^, vs. ≤15 mmHg group			0.0469 *	0.0493 *	<0.0001 **	
*p*-value ^‡^, vs. >15 and ≤18 mmHg group				0.9990	0.0017 **	
*p*-value ^‡^, vs. >18 and ≤21 mmHg group					0.0007 **	
*p* value ^#^, pre- vs. post-op	<0.0001 **					
						
Medication						
Pre-op	2.7 ± 1.2 (0, 5)	2.6 ± 1.2 (1, 5)	2.4 ± 1.2 (0, 4)	2.9 ± 1.1 (1, 5)	2.7 ± 1.1 (1, 4)	0.2499
12 M post-op	2.3 ± 1.0 (0, 4)	2.3 ± 1.0 (0, 4)	2.1 ± 1.1 (0, 4)	2.6 ± 0.9 (1, 4)	2.2 ± 0.9 (0, 3)	0.1966
Difference	0.4	0.3	0.3	0.3	0.5	
*p* value ^#^, pre- vs. post-op	<0.0001 **	0.0392 *	0.0180 *	0.0318 *	0.0774	
%Medication reduction, %	3.0 ± 47.7 (−200, +100)	0.8 ± 48.2 (−200, 100)	9.0 ± 22.0 (−50, 66.7)	0.7 ± 53.0 (−200, 66.7)	3.0 ± 60.7 (−200, 100)	0.9063

Data are expressed in mean ± SD (range). ^†^ *p* values are calculated using one−way analysis of variance (ANOVA) among 4 groups stratified by preoperative intraocular pressure level (i.e., Q1-Q4). ^‡^ if ANOVA is significant (*p* < 0.05), Tukey−Kramer honesty significant difference tests are used for each pair comparison (i.e., Q1-Q4). ^#^ *p* values are calculated by using paired *t*-test between pre-operative and 12 months post-operative values. * and ** indicate significance levels of 5% and 1%, respectively. Abbreviations: IOP, intraocular pressure; %IOP reduction, (preoperative IOP minus 12 months IOP)/preoperative IOP ∗ 100; %medication reduction, (preoperative medication minus 12 months medication)/preoperative medication ∗ 100.

**Table 3:** Intraoperative and postoperative complications and interventions.

Complications, *n* (%)		Interventions, *n* (%)	
Intraoperative		Intraoperative	
Iris prolapse, IFIS	4 (3)	CTR implantation	2 (2)
Angle recession	1 (<1)	Goniocynechialysis	1 (<1)
Any complication	5 (4)	Any intervention	3 (2)
Postoperative		Postoperative	
Layered hyphema	42 (33)	Hyphema washout	9 (7)
Transient IOP elevation >30 mmHg	6 (5)	Posterior synechialysis, pupiloplasty	2 (2)
Macular edema	5 (4)	Pars-plana vitrectomy	2 (2)
Fibrin formation in anterior chamber	3 (2)	Anterior chamber injection of tPA	1 (<1)
Posterior synechia, corectopia	2 (2)	Sub-Tenon triamcinolone injection	1 (<1)
Vitreous hemorrhage	2 (<1)	Intravitreal anti-VEGF injection	1 (<1)
Cataract	1 (<1)	Nd:YAG laser capsulotomy	1 (<1)
Persistent hypotony	1 (<1)	Anterior chamber OVD injection	1 (<1)
Iritis	1 (<1)	Incision of CCC edge by Nd:YAG laser	1 (<1)
After cataract	1 (<1)		
Contraction of CCC edge	1 (<1)		
Age-related macular degeneration	1 (<1)		
Any complication	57 (45)	Any intervention	16 (13)

Abbreviations: IFIS, intraoperative floppy iris syndrome; IOP, intraoperative pressure; CCC, continuous curvilinear capsulorrhexis; CTR, capsular tension ring; tPA, tissue plasminogen activator; VEGF, vascular endothelial growth factor; Nd: YAG, neodymium:yttrium-aluminium-garnet; OVD, ocular viscoelastic device.

**Table 4:** Assessment of factors associated with postoperative intraocular pressure levels.

Parameters	r (95% CI Range)	Standard β	*p* Value
Age (/year)	−0.08 (−0.14, −0.01)	−0.22	0.0283 *
Female (/male)	0.15 (−0.46, 0.76)	0.04	0.6268
EXG (/POAG)	−0.17 (−0.90, 0.56)	−0.04	0.6444
µLOT alone (/combined µLOT + cataract surgery)	0.17 (−0.90, 1.23)	0.03	0.7566
Phakic eye (/pseudophakic eye)	−0.68 (−2.32, 0.96)	−0.09	0.4099
Extent of trabeculotomy (/clock hours)	0.18 (−0.32, 0.69)	0.06	0.4695
Preoperative IOP (/mmHg)	0.33 (0.22, 0.44)	0.51	<0.0001 **
Preoperative number of medications (/medication)	0.09 (−0.47, 0.64)	0.03	0.7611

Possible associations between IOP at final visit and various parameters are assessed using multiple regression analysis. * and ** indicate significance levels of 5% and 1%, respectively. Abbreviations: POAG, primary open-angle glaucoma; EXG, exfoliation glaucoma; µLOT, microhook *ab interno* trabeculotomy; r, regression coefficient; CI, confidence interval.

**Table 5:** Success rate of IOP control at 12 months postoperatively.

Parameters	Total	Q1	Q2	Q3	Q4	*p* Value ^†^
IOP ≤18 mmHg						
Success, *n* (%)	119 (94)	38 (100)	30 (100)	34 (94)	17 (77)	0.0003 **
Failure, *n* (%)	7 (6)	0 (0)	0 (0)	2 (6)	5 (23)	
IOP ≤15 mmHg						
Success, *n* (%)	105 (83)	37 (97)	27 (90)	24 (67)	17 (77)	0.0025 **
Failure, *n* (%)	21 (17)	1 (3)	3 (10)	12 (33)	5 (23)	
IOP ≤ 12 mmHg						
Success, *n* (%)	55 (44)	20 (53)	15 (50)	10 (28)	10 (45)	0.1869
Failure, *n* (%)	71 (56)	18 (47)	15 (50)	26 (72)	12 (55)	
IOP reduction ≥ 20%						
Success, *n* (%)	77 (61)	15 (39)	17 (57)	27 (75)	18 (82)	0.0002 **
Failure, *n* (%)	49 (39)	23 (61)	13 (43)	9 (25)	4 (18)	
IOP reduction ≥ 0%						
Success, *n* (%)	116 (92)	34 (89)	29 (97)	34 (94)	19 (86)	0.8393
Failure, *n* (%)	10 (8)	4 (11)	1 (3)	2 (6)	3 (14)	
IOP ≤ 18 mmHg and IOP reduction ≥ 20%						
Success, *n* (%)	76 (60)	15 (39)	17 (57)	27 (75)	17 (77)	0.0005 **
Failure, *n* (%)	50 (40)	23 (61)	13 (43)	9 (25)	5 (23)	
IOP ≤ 15 mmHg and IOP reduction ≥ 20%						
Success, *n* (%)	73 (58)	15 (39)	17 (57)	24 (67)	17 (77)	0.0020 **
Failure, *n* (%)	53 (42)	23 (61)	13 (43)	12 (33)	5 (23)	
IOP ≤ 12 mmHg and IOP reduction ≥ 20%						
Success, *n* (%)	50 (40)	15 (39)	15 (50)	10 (28)	10 (45)	0.8026
Failure, *n* (%)	76 (60)	23 (61)	15 (50)	26 (72)	12 (55)	
IOP ≤ 18 mmHg and IOP reduction ≥ 0%						
Success, *n* (%)	110 (87)	34 (89)	29 (97)	32 (89)	15 (68)	0.0324 *
Failure, *n* (%)	16 (13)	4 (11)	1 (3)	4 (11)	7 (32)	
IOP ≤ 15 mmHg and IOP reduction ≥ 0%						
Success, *n* (%)	98 (78)	34 (89)	26 (87)	23 (64)	15 (68)	0.0069 **
Failure, *n* (%)	28 (22)	4 (11)	4 (13)	13 (36)	7 (32)	
IOP ≤ 12 mmHg and IOP reduction ≥ 0%						
Success, *n* (%)	52 (41)	19 (50)	15 (50)	9 (25)	9 (41)	0.1192
Failure, *n* (%)	74 (59)	19 (50)	15 (50)	27 (75)	13 (59)	

^†^*p* values are calculated using exact Cochrane−Armitage trend test among 4 groups stratified by preoperative intraocular pressure level. * and ** indicate significance level of 5% and 1%, respectively. Abbreviations: IOP, intraocular pressure.

## Data Availability

Data is fully available upon reasonable request to corresponding author.

## Supplementary Materials

The following are available online at https://www.mdpi.com/article/10.3390/jcm10153327/s1, Supplementary Table S1. Data and list of literatures used for generation of Figure 1.
