# Supplementary material for: Effects of Preoperative Intraocular Pressure Level on Surgical Results of Microhook Ab Interno Trabeculotomy
Source: J Clin Med. 2021 Jul 28;10(15):3327. doi: 10.3390/jcm10153327 (PMC8348769; doi:10.3390/jcm10153327)
Supplement: Supplementary file 1 [file jcm-10-03327-s001.zip › jcm-1303478-supplementary.pdf]

Supplementary Table S1: Pre- and 12 months post-operative IOPs in current and previous studies of ab interno goniotomy procedures reported in subjects group including POAG

Procedure	Combined CE	Preoperative IOP (mmHg)	Postoperative IOP (mmHg)	Difference (mmHg)	%IOP reduction	Reference
μLOT	mix	18.8	12.9	5.9	31	Current study
μLOT	combined	19.3	12.8	6.5	34	[1]
μLOT	solo	22.4	14.4	8	36	[1]
μLOT	mix	28.5	15	13.5	47	[2]
μLOT	mix	27	16	11	41	[2]
μLOT	combined	20.5	13.9	6.6	32	[3]
μLOT	combined	24.7	13	11.7	47	[4]
μLOT	mix	24.1	12.5	11.6	48	[5]
μLOT	mix	28.4	17.8	10.6	37	[6]
KDB	combined	22.3	13.6	8.7	39	[3]
KDB	combined	23.2	16.7	6.5	28	[4]
KDB	combined	24	17.2	6.8	28	[7]
KDB	combined	14.8	13.1	1.7	11	[8]
KDB	combined	21.3	13.5	7.8	37	[8]
KDB	solo	21.6	16.5	5.1	24	[9]
KDB	combined	19.8	13.4	6.4	32	[10]
KDB	combined	16.5	14.1	2.4	15	[11]
KDB	solo	24.3	16.9	7.4	30	[11]
KDB	combined	18.5	15.4	3.1	17	[12]
KDB	mix	17.1	14.7	2.4	14	[13]
KDB	combined	18.2	13.2	5	27	[14]
KDB	combined	17.4	15	2.4	14	[15]
KDB	combined	16.8	12.4	4.4	26	[16]
KDB	combined	16.7	13.8	2.9	17	[17]
KDB	solo	20.4	14.1	6.3	31	[17]

GATT	mix	18.7	13.8	4.9	26	[18]
GATT	mix	18.7	13.9	4.8	26	[18]
GATT	mix	18.3	13.3	5	27	[18]
GATT	mix	28.3	13.3	15	53	[19]
GATT	combined	22.7	13.5	9.2	41	[20]
GATT	solo	24.9	14.3	10.6	43	[20]
GATT	solo	27.4	14.4	13	47	[21]
GATT	combined	24.7	16.3	8.4	34	[22]
GATT	solo	26	16.4	9.6	37	[22]
GATT	combined	21.9	12	9.9	45	[23]
GATT	solo	26.6	14.7	11.9	45	[23]
GATT	combined	23.9	15.5	8.4	35	[24]
GATT	solo	25.6	15.7	9.9	39	[24]
TOM	mix	24.6	13.3	11.3	46	[5]
TOM	mix	15.3	12.5	2.8	18	[25]
TOM	mix	22	13	9	41	[25]
TOM	mix	31.9	13.5	18.4	58	[25]
TOM	mix	22.3	16	6.3	28	[26]
TOM	combined	18.3	13.3	5	27	[27]
TOM	combined	15.3	13.3	2	13	[28]
TOM	combined	19.3	13.8	5.5	28	[29]
TOM	mix	23	16.8	6.2	27	[30]
TOM	combined	20	16.8	3.2	16	[31]
TOM	mix	20	13.6	6.4	32	[32]
TOM	mix	21.2	16.1	5.1	24	[33]
TOM	mix	21.2	15.7	5.5	26	[33]
TOM	mix	21.4	16.5	4.9	23	[34]
TOM	mix	24	16.1	7.9	33	[35]
TOM	mix	22.6	15.7	6.9	31	[35]
TOM	mix	23.6	15	8.6	36	[36]

TOM	mix	22.6	15.8	6.8	30	[37]
TOM	solo	19.1	14.4	4.7	25	[38]
TOM	solo	22.5	13.7	8.8	39	[38]
TOM	mix	23.6	16	7.6	32	[39]
TOM	mix	37.6	16.9	20.7	55	[40]
TOM	combined	18.3	13.5	4.8	26	[41]
TOM	combined	18.1	15.2	2.9	16	[42]
TOM	combined	19.3	15.6	3.7	19	[42]
TOM	combined	19.2	15.1	4.1	21	[42]
TOM	combined	25.7	16.4	9.3	36	[42]
TOM	combined	20.9	16	4.9	23	[43]
TOM	combined	22.3	15.6	6.7	30	[44]
TOM	mix	19.6	15.7	3.9	20	[45]
TOM	mix	21.6	15.9	5.7	26	[45]
TOM	mix	24.5	16.2	8.3	34	[45]
TOM	mix	29	16.7	12.3	42	[45]
TOM	solo	20.7	16.2	4.5	22	[46]
TOM	solo	23	16.4	6.6	29	[46]
TOM	solo	26.1	16.6	9.5	36	[46]
TOM	solo	29.1	16.7	12.4	43	[46]
TOM	mix	31.6	16.4	15.2	48	[47]
TOM	solo	21.7	15.9	5.8	27	[48]
TOM	combined	19.7	15.5	4.2	21	[48]
TOM	solo	28.8	17.1	11.7	41	[49]
TOM	solo	22.3	15.1	7.2	32	[50]
TOM	mix	19.3	14.6	4.7	24	[51]
TOM	solo	27.3	15.7	11.6	42	[52]
TOM	solo	26.1	16.4	9.7	37	[52]
TOM	combined	20.7	15.7	5	24	[52]
TOM	combined	22.6	17	5.6	25	[52]

TOM	solo	25.9	19.3	6.6	25	[53]
TOM	combined	16.5	14.6	1.9	12	[53]
TOM	solo	25.5	18	7.5	29	[54]
TOM	combined	19.9	15.8	4.1	21	[54]
TOM	combined	20	14.8	5.2	26	[55]
TOM	mix	25.7	16.1	9.6	37	[56]
TOM	mix	27.6	16.4	11.2	41	[57]
TOM	solo	28.2	16.3	11.9	42	[58]

IOP, intraocular pressure; POAG, primary open angle glaucoma; μLOT, microhook trabeculotomy; KDB, Kahook Dual Blade; GATT, gonioscopy-assisted transluminal trabeculotomy; TOM, Trabectome; CE, cataract extraction.

Reference

1. Tanito M, Sugihara K, Tsutsui A, et al. Midterm Results of Microhook ab Interno Trabeculotomy in Initial 560 Eyes with Glaucoma. J Clin Med 2021;10(4).
2. Mori S, Murai Y, Ueda K, et al. Comparison of efficacy and early surgery-related complications between one-quadrant and two-quadrant microhook ab interno trabeculotomy: a propensity score matched study. Acta Ophthalmol 2021.
3. Omoto T, Fujishiro T, Asano-Shimizu K, et al. Comparison of 12-month surgical outcomes of ab interno trabeculotomy with phacoemulsification between spatula-shaped and dual-blade microhooks. Jpn J Ophthalmol 2021.
4. Aoki R, Hirooka K, Goda E, et al. Comparison of Surgical Outcomes Between Microhook Ab Interno Trabeculotomy and Goniotomy with the Kahook Dual Blade in Combination with Phacoemulsification: A Retrospective, Comparative Case Series. Adv Ther 2021;38(1):329-36.
5. Tojo N, Otsuka M, Hayashi A. Comparison of trabectome and microhook surgical outcomes. Int Ophthalmol 2021;41(1):21-6.
6. Mori S, Murai Y, Ueda K, et al. A comparison of the 1-year surgical outcomes of ab externo trabeculotomy and microhook ab interno trabeculotomy using propensity score analysis. BMJ Open Ophthalmol 2020;5(1):e000446.
7. Chihara E, Chihara T. Turn Back Elevation of Once Reduced IOP After Trabeculotomy Ab Externo and Kahook Dual Blade Surgeries Combined with Cataract Surgery. Clin Ophthalmol 2020;14:4359-68.
8. Porter M, Garza A, Gallardo M. Excisional Goniotomy in Latino Patients with Open-Angle Glaucoma: Outcomes Through 24 Months. Clin Ophthalmol 2020;14:3619-25.

9. ElMallah MK, Berdahl JP, Williamson BK, et al. Twelve-Month Outcomes of Stand-Alone Excisional Goniotomy in Mild to Severe Glaucoma. Clin Ophthalmol 2020;14:1891-7.
10. Iwasaki K, Takamura Y, Orii Y, et al. Performances of glaucoma operations with Kahook Dual Blade or iStent combined with phacoemulsification in Japanese open angle glaucoma patients. Int J Ophthalmol 2020;13(6):941-5.
11. Wakil SM, Birnbaum F, Vu DM, et al. Efficacy and safety of a single-use dual blade goniotomy: 18-month results. J Cataract Refract Surg 2020;46(10):1408-15.
12. Falkenberry S, Singh IP, Crane CJ, et al. Excisional goniotomy vs trabecular microbypass stent implantation: a prospective randomized clinical trial in eyes with mild to moderate open-angle glaucoma. J Cataract Refract Surg 2020;46(8):1165-71.
13. Kornmann HL, Fellman RL, Feuer WJ, et al. Early Results of Goniotomy with the Kahook Dual Blade, a Novel Device for the Treatment of Glaucoma. Clin Ophthalmol 2019;13:2369-76.
14. ElMallah MK, Seibold LK, Kahook MY, et al. 12-Month Retrospective Comparison of Kahook Dual Blade Excisional Goniotomy with Istent Trabecular Bypass Device Implantation in Glaucomatous Eyes at the Time of Cataract Surgery. Adv Ther 2019;36(9):2515-27.
15. Le C, Kazaryan S, Hubbell M, et al. Surgical Outcomes of Phacoemulsification Followed by iStent Implantation Versus Goniotomy With the Kahook Dual Blade in Patients With Mild Primary Open-angle Glaucoma With a Minimum of 12-Month Follow-up. J Glaucoma 2019;28(5):411-4.
16. Dorairaj SK, Seibold LK, Radcliffe NM, et al. 12-Month Outcomes of Goniotomy Performed Using the Kahook Dual Blade Combined with Cataract Surgery in Eyes with Medically Treated Glaucoma. Adv Ther 2018;35(9):1460-9.
17. Sieck EG, Epstein RS, Kennedy JB, et al. Outcomes of Kahook Dual Blade Goniotomy with and without Phacoemulsification Cataract Extraction. Ophthalmol Glaucoma 2018;1(1):75-81.
18. Sato T, Kawaji T. 12-month randomised trial of 360° and 180° Schlemm's canal incisions in suture trabeculotomy ab interno for open-angle glaucoma. Br J Ophthalmol 2020.
19. Yalinbas D, Dilekmen N, Hepsen IF. Comparison of Ab Externo and Ab Interno 360-degree Suture Trabeculotomy in Adult Open-angle Glaucoma. J Glaucoma 2020;29(11):1088-94.
20. Aktas Z, Ucgul AY, Bektas C, Sahin Karamert S. Surgical Outcomes of Prolene Gonioscopy-assisted Transluminal Trabeculotomy in Patients With Moderate to Advanced Open-Angle Glaucoma. J Glaucoma 2019;28(10):884-8.
21. Aktas Z, Ozmen MC, Atalay HT, Ucgul AY. Evaluation of episcleral venous fluid wave during gonioscopy assisted transluminal trabeculotomy in patients with advanced glaucoma. Eye (Lond) 2019;33(4):668-73.

22. Grover DS, Smith O, Fellman RL, et al. Gonioscopy-assisted Transluminal Trabeculotomy: An Ab Interno Circumferential Trabeculotomy: 24 Months Follow-up. J Glaucoma 2018;27(5):393-401.

23. Rahmatnejad K, Pruzan NL, Amanullah S, et al. Surgical Outcomes of Gonioscopy-assisted Transluminal Trabeculotomy (GATT) in Patients With Open-angle Glaucoma. J Glaucoma 2017;26(12):1137-43.

24. Grover DS, Godfrey DG, Smith O, et al. Gonioscopy-assisted transluminal trabeculotomy, ab interno trabeculotomy: technique report and preliminary results. Ophthalmology 2014;121(4):855-61.

25. Tojo N, Hayashi A. The Outcomes of Trabectome Surgery in Patients with Low, Middle, and High Preoperative Intraocular Pressure. Clin Ophthalmol 2020;14:4099-108.

26. Strzalkowska A, Strzalkowski P, Al Yousef Y, et al. Exact matching of trabectome-mediated ab interno trabeculectomy to conventional trabeculectomy with mitomycin C followed for 2 years. Graefes Arch Clin Exp Ophthalmol 2020.

27. Al Yousef Y, Strzalkowska A, Hillenkamp J, et al. Comparison of a second-generation trabecular bypass (iStent inject) to ab interno trabeculectomy (Trabectome) by exact matching. Graefes Arch Clin Exp Ophthalmol 2020;258(12):2775-80.

28. Weiner AJ, Weiner Y, Weiner A. Intraocular Pressure After Cataract Surgery Combined With Ab Interno Trabeculectomy Versus Trabecular Micro-bypass Stent: An Intrasubject Same-surgeon Comparison. J Glaucoma 2020;29(9):773-82.

29. Jozic L, Magner J, Funk J, Töteberg-Harms M. Success of combined cataract extraction plus excimer laser trabeculotomy exceeds that of combined ab interno trabeculectomy with the trabectome or cataract extraction alone. Int Ophthalmol 2020;40(3):529-37.

30. Avar M, Jordan JF, Neuburger M, et al. Long-term follow-up of intraocular pressure and pressure-lowering medication in patients after ab-interno trabeculectomy with the Trabectome. Graefes Arch Clin Exp Ophthalmol 2019;257(5):997-1003.

31. Ting JLM, Rudnisky CJ, Damji KF. Prospective randomized controlled trial of phaco-trabectome versus phaco-trabeculectomy in patients with open angle glaucoma. Can J Ophthalmol 2018;53(6):588-94.

32. Esfandiari H, Shah P, Torkian P, et al. Five-year clinical outcomes of combined phacoemulsification and trabectome surgery at a single glaucoma center. Graefes Arch Clin Exp Ophthalmol 2019;257(2):357-62.

33. Nazarali SA, Damji KF. Ab interno trabeculectomy with Trabectome: outcomes in African American versus Caucasian patients. Can J Ophthalmol 2018;53(4):361-4.

34. Dang YL, Wang X, Dai WW, et al. Two-year outcomes of ab interno trabeculectomy with the Trabectome for Chinese primary open angle glaucoma: a retrospective multicenter study. Int J Ophthalmol 2018;11(6):945-50.

35. Ahmed SF, Bhatt A, Schmutz M, Mosaed S. Trabectome outcomes across the spectrum of glaucoma disease severity. Graefes Arch Clin Exp Ophthalmol 2018;256(9):1703-10.

36. Esfandiari H, Shazly TA, Waxman SA, et al. Similar Performance of Trabectome and Ahmed Glaucoma Devices in a Propensity Score-matched Comparison. J Glaucoma 2018;27(6):490-5.

37. Kinoshita-Nakano E, Nakanishi H, Ohashi-Ikeda H, et al. Comparative outcomes of trabeculotomy ab externo versus trabecular ablation ab interno for open angle glaucoma. Jpn J Ophthalmol 2018;62(2):201-8.

38. Pahlitzsch M, Davids AM, Zorn M, et al. Three-year results of ab interno trabeculectomy (Trabectome): Berlin study group. Graefes Arch Clin Exp Ophthalmol 2018;256(3):611-9.

39. Okeke CO, Miller-Ellis E, Rojas M. Trabectome success factors. Medicine (Baltimore) 2017;96(24):e7061.

40. Akil H, Chopra V, Huang AS, et al. Short-Term Clinical Results of Ab Interno Trabeculotomy Using the Trabectome with or without Cataract Surgery for Open-Angle Glaucoma Patients of High Intraocular Pressure. J Ophthalmol 2017;2017:8248710.

41. Hashemian SJ, Miraftabi A, Jafari ME, Hemami MR. Combined cataract extraction and trabeculotomy by the internal approach for coexisting cataract and open-angle glaucoma. J Curr Ophthalmol 2017;29(1):17-22.

42. Roy P, Loewen RT, Dang Y, et al. Stratification of phaco-trabectome surgery results using a glaucoma severity index in a retrospective analysis. BMC Ophthalmol 2017;17(1):30.

43. Kurji K, Rudnisky CJ, Rayat JS, et al. Phaco-trabectome versus phaco-iStent in patients with open-angle glaucoma. Can J Ophthalmol 2017;52(1):99-106.

44. Gonnermann J, Bertelmann E, Pahlitzsch M, et al. Contralateral eye comparison study in MICS & MIGS: Trabectome® vs. iStent inject®. Graefes Arch Clin Exp Ophthalmol 2017;255(2):359-65.

45. Dang Y, Roy P, Bussel, II, et al. Combined analysis of trabectome and phaco-trabectome outcomes by glaucoma severity. F1000Res 2016;5:762.

46. Loewen RT, Roy P, Parikh HA, et al. Impact of a Glaucoma Severity Index on Results of Trabectome Surgery: Larger Pressure Reduction in More Severe Glaucoma. PLoS One 2016;11(3):e0151926.

47. Shoji N, Kasahara M, Iijima A, et al. Short-term evaluation of Trabectome surgery performed on Japanese patients with open-angle glaucoma. Jpn J Ophthalmol 2016;60(3):156-65.

48. Parikh HA, Bussel, II, Schuman JS, et al. Coarsened Exact Matching of Phaco-Trabectome to Trabectome in Phakic Patients: Lack of Additional Pressure Reduction from Phacoemulsification. PLoS One 2016;11(2):e0149384.

49. Yildirim Y, Kar T, Duzgun E, et al. Evaluation of the long-term results of trabectome surgery. Int Ophthalmol 2016;36(5):719-26.

50. Mizoguchi T, Nishigaki S, Sato T, et al. Clinical results of Trabectome surgery for open-angle glaucoma. Clin Ophthalmol 2015;9:1889-94.

51. Fellman RL, Feuer WJ, Grover DS. Episcleral Venous Fluid Wave Correlates with Trabectome Outcomes: Intraoperative Evaluation of the Trabecular Outflow Pathway. Ophthalmology 2015;122(12):2385-91.e1.

52. Bussel, II, Kaplowitz K, Schuman JS, Loewen NA. Outcomes of ab interno trabeculectomy with the trabectome by degree of angle opening. Br J Ophthalmol 2015;99(7):914-9.

53. Ahuja Y, Ma Khin Pyi S, Malihi M, et al. Clinical results of ab interno trabeculotomy using the trabectome for open-angle glaucoma: the Mayo Clinic series in Rochester, Minnesota. Am J Ophthalmol 2013;156(5):927-35.e2.

54. Ting JL, Damji KF, Stiles MC. Ab interno trabeculectomy: outcomes in exfoliation versus primary open-angle glaucoma. J Cataract Refract Surg 2012;38(2):315-23.

55. Francis BA, Minckler D, Dustin L, et al. Combined cataract extraction and trabeculotomy by the internal approach for coexisting cataract and open-angle glaucoma: initial results. J Cataract Refract Surg 2008;34(7):1096-103.

56. Minckler D, Mosaed S, Dustin L, Ms BF. Trabectome (trabeculectomy-internal approach): additional experience and extended follow-up. Trans Am Ophthalmol Soc 2008;106:149-59; discussion 59-60.

57. Minckler D, Baerveldt G, Ramirez MA, et al. Clinical results with the Trabectome, a novel surgical device for treatment of open-angle glaucoma. Trans Am Ophthalmol Soc 2006;104:40-50.

58. Minckler DS, Baerveldt G, Alfaro MR, Francis BA. Clinical results with the Trabectome for treatment of open-angle glaucoma. Ophthalmology 2005;112(6):962-7.
